# Preliminary results on an autochthonous dengue outbreak in Lombardy Region, Italy, August 2023

**DOI:** 10.2807/1560-7917.ES.2023.28.37.2300471

**Published:** 2023-09-14

**Authors:** Irene Cassaniti, Guglielmo Ferrari, Sabrina Senatore, Eva Rossetti, Francesco Defilippo, Manuel Maffeo, Luigi Vezzosi, Giulia Campanini, Antonella Sarasini, Stefania Paolucci, Antonio Piralla, Davide Lelli, Ana Moreno, Maira Bonini, Marcello Tirani, Lorenzo Cerutti, Stefano Paglia, Angelo Regazzetti, Marco Farioli, Antonio Lavazza, Marino Faccini, Francesca Rovida, Danilo Cereda, Fausto Baldanti, Lombardy Dengue network, Lombardy Dengue Network, Daniele Lilleri, Milena Furione, Federica Zavaglio, Maya Carrera, Giuditta Scardina, Marzia Soresini, Manuela Barozzi, Rita Brugnoli, Nunzia Laini, Francesca Bonalda, Sara Arfani, Giuditta Zamboni, Manuela Piazza, Fanny Delfanti, Piergiuseppe Ferrari, Anxhela Dafa, Antonella Negri, Filippa Parisi, Michela Viscardi, Federica Attanasi, Giovanni Manarolla, Mario Chiari, Enrico Tallarita

**Affiliations:** 1 Department of Clinical, Surgical, Diagnostic and Paediatric Sciences, University of Pavia, Pavia, Italy; 2 SC Microbiology and Virology, IRCCS Policlinico San Matteo, Pavia, Italy; 3 Department of Hygiene and Health Prevention, Health Protection Agency, Metropolitan Area of Milan, Milan, Italy; 4 Virology Department, Istituto Zooprofilattico Sperimentale della Lombardia ed Emilia Romagna, Brescia, Italy; 5 Postgraduate School in Public Health, Department Biomedical Sciences for Health, University of Milan, Milan, Italy; 6 Department of Hygiene and Health Prevention, Health Protection Agency Val Padana, Mantova, Italy; 7 General Directorate of Welfare, Regione Lombardia, Milan, Italy; 8 Health Director Staff, Health Protection Agency, Metropolitan Area of Milan, Milan, Italy; 9 SC Chemical-Clinical Analysis and Microbiology Laboratory, ASST Lodi, Lodi, Italy; 10 Department of Emergency and Urgency, ASST Lodi, Lodi, Italy; 11 SC Infectious and Tropical Diseases, ASST Lodi, Lodi, Italy; 12 The members of the network are listed under Collaborators; * These authors contributed equally to this work and share first authorship.; ** These authors contributed equally to this work and share last authorship.

**Keywords:** Dengue, phylogenetic analysis, serology, autochthonous cases, Italy, virus

## Abstract

In August 2023, six locally acquired dengue virus 1 infections were detected in Lodi province, Lombardy Region, in northern Italy, where the vector *Aedes albopictus* is present. Four cases were hospitalised, none died. The viruses clustered with Peruvian and Brazilian strains collected between 2021 and 2023. This preliminary report highlights the importance of continued integrated surveillance of imported vector-borne virus infections and the potential for tropical disease outbreaks in highly populated regions of northern Italy where competent vectors are present.

Dengue virus (DENV) infection has become a growing health concern worldwide [1,2]. In the last decades, few autochthonous cases and limited outbreaks have been reported in Europe [3-7]. Here, we describe an outbreak of six autochthonous dengue cases occurring in Lodi province, Lombardy Region in northern Italy in August 2023.

## Case description and sampling

On 3 August, an individual (Case 1) living in a village of ca 4,500 inhabitants in the Lodi province was admitted to the emergency department of the local hospital with fever (> 39 °C), arthralgia, myalgia, maculopapular rash, confusion and headache. The patient was first discharged with supportive treatment. Six days after symptoms onset, the patient was hospitalised because symptoms persisted. The clinical samples were referred to the Microbiology and Virology Unit of Fondazione IRCCS Policlinico San Matteo, Pavia, for suspected infection with West Nile virus (WNV) on 9 August 2023. Samples were tested for WNV-specific antibodies using West Nile Virus VirClia IgM monotest and West Nile Virus VirClia IgG monotest (VirCell Microbiologists, Spain). Virological investigations were performed with a pan-flavivirus heminested RT-PCR [8] and a WNV-specific RT-PCR [9] on plasma and urine samples.

The pan-flavivirus heminested RT-PCR resulted positive in plasma and urine, while the WNV-specific antibody test and RT-PCR were both negative. A subsequent sequencing analysis revealed the presence of DENV serotype 1 RNA. The diagnosis of DENV infection was confirmed by the presence of viral RNA in plasma and urine by a DENV-specific RT-PCR [10] and detection of DENV IgM antibodies (dengue VirClia IgM monotest and dengue VirClia IgG monotest, VirCell Microbiologists). Further, IgG seroconversion was documented in a second sample, collected after 15 days (Table). Following the DENV diagnosis of Case 1, between 22 and 25 August, five additional people living in a neighbouring area of the same village of the first case were referred to the local hospital for suspected DENV infection and confirmed as for Case 1. Their details are summarised in the Table. The six cases were living at a maximum linear distance of 450 m from each other. The first case did not share outdoor activities with the other dengue cases. All six patients had symptoms including fever, arthralgia, myalgia and headache and note had travelled in the past months. At the moment of writing, all six cases were alive.

**Table:** Clinical and virological data of dengue cases, Italy, August 2023 (n = 6)

Demographic and clinical characteristics				Antibody (index)		Pan-flavivirus PCR		DENV-specific RT-PCR (copies/mL)		Sequencing
Case	Hospitalisation	Days from symptom onset to sampling	Sample date	IgM	IgG	Plasma	Urine	Plasma	Urine	Typing
1	Yes	6	9 Aug	12.5	< 0.9	Positive	Positive	3.5 × 10^5^	2,025	DENV-1
		20	23 Aug	34.5	1.3	Negative	Positive	< 45	< 45	
2	No	18	22 Aug	32.2	1.6	Negative	Positive	< 45	990	NA
3	Yes	6	22 Aug	12.9	< 0.9	Positive	Negative	2.3 × 10^6^	< 45	DENV-1
4	Yes	2	23 Aug	< 0.9	< 0.9	Positive	Negative	15 × 10^6^	< 45	DENV-1
5	No	4	25 Aug	3.8	< 0.9	Positive	Positive	6.4 × 10^5^	630	DENV-1
6	Yes	6	25 Aug	25.7	< 0.9	Positive	Positive	1 × 10^5^	< 45	DENV-1

DENV: dengue virus; NA: not available; RT-PCR: reverse transcription PCR.

Antibody index was considered negative when < 0.9 and positive when > 1.1; DENV-specific RT-PCR was considered negative when < 45 copies/mL and positive when ≥ 45 copies/mL.

## Sequencing

Whole genome sequencing (WGS) was performed using the metagenomic approach as previously described by Piralla et al. [11]. Reads were mapped to the genome ON123600 using the INSaFLU pipeline (https://insaflu.insa.pt) [12]. Phylogenetic analysis was performed on WGS obtained directly from clinical samples of five dengue cases (GenBank accession numbers: OR512925-OR512929) (Figure). A maximum likelihood phylogenetic tree was inferred using the IQ-TREE web server (v1.6.8) [13], the robustness of branches was evaluated using the Shimodaira–Hasegawa approximate likelihood-ratio test (SH-aLRT) and ultrafast bootstrap approximation tests. The Italian strains clustered with Peruvian and Brazilian strains collected in the period 2021 to 2023, with an average nucleotide identity of 98.6% (range: 95.1–99.9) between DENV-1 strains of genotype V.

**Figure:**
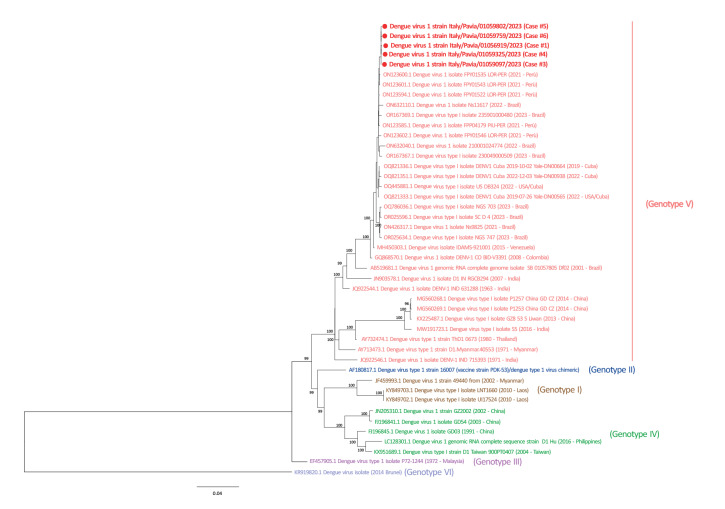
Phylogenetic tree of dengue virus serotype 1 complete genome sequences, Italy, August 2023 (n = 5 samples from the current outbreak)

DENV: dengue virus.

Sequences from the cases in this manuscript are shown in bold. Reference sequences were obtained from GenBank.

## Public health measures

When the first dengue case, defined according with the Commission Implementing Decision (EU) [14], was notified to the Local Health Authority (ATS) on 18 August 2023, ATS immediately carried out the epidemiological investigation. The patient reported that they had not travelled abroad in the weeks before the onset of symptoms and not had contact with people who had travelled to DENV-endemic areas. In addition, no travel-associated cases were reported in the area. Thus, Case 1 was classified as autochthonous and immediate notification was given by the ATS to the Regional and National Health Authorities. During the epidemiological investigations, Case 1 stated that they spent many hours a day outdoors, thus a careful mapping of the frequented places was carried out. Disinfestation activities for mosquito control (Cipermetrin and Permofen Forte; 1R-trans Phenothrin and Tetramethrin) were carried out around their home and in the areas they frequented. The neighbours of Case 1 and subsequent dengue cases were informed on mosquito prevention and control. Disinfestation was performed for each case as described above.

Hospitals and physicians in the Lodi province were notified to remain alert for patients with possible dengue symptoms. The population of the village where DENV cases were detected was informed about DENV.

## Entomological investigations

According to the Italian *National Plan for Prevention, Surveillance and Response to* *Arboviruses* [15], specific entomological inspections were carried out within a radius of 200 m around the homes and places frequented by the diagnosed dengue cases. Inspection of the home address was conducted within 2 days of the notification of each suspected or confirmed case. Adult mosquitoes were captured using different traps. The captured mosquitoes were immediately sent refrigerated to the laboratory at Istituto Zooprofilattico Sperimentale della Lombardia e Emilia-Romagna (IZSLER). Mosquitoes were morphologically identified using taxonomic keys [16] and pooled according to the date and site of collection.

These entomological inspections were performed 13 days after symptom onset of Case 1. By 30 August, 131 *Aedes albopictus* females had been sampled, divided into eight pools and processed for flavivirus genome detection. The mosquito pools were homogenised, viral RNA was extracted and analysed with the same pan-flavivirus RT-PCR assay as used for the human samples [8]. To date, all tested mosquito pools analysed were negative. More samplings are scheduled at different sites until 7 September, close to the homes of the other five dengue cases.

## Discussion 

DENV infections have been reported in more than 100 countries [17], the vast majority in Asia, South Pacific Islands, the Carribbean and Latin America [18]. However, in the last decades, an increasing number of autochthonous cases have occurred in European countries were competent *Aedes* mosquitos are present. In 2022, 65 autochthonous dengue cases were reported in France [19]. In Italy, *Aedes albopictus* was reported for the first time in the 1990s [20] and it is likely that it has been present in the Lombardy Region in the Lodi province since the mid-1990s [21]. The vector is today present throughout Italy, and the first documented outbreak of autochthonous dengue occurred in the Veneto Region in 2020 [6,22]. Moreover, at the moment of writing, three autochthonous cases of DENV infection have been also reported in central Italy [23].

Effective surveillance systems for imported and autochthonous arboviruses infection as well as vector control programmes are active in Lombardy Region, in accordance with the Italian *National Plan for Prevention, Surveillance and Response to* *Arboviruses* [15]. Timely reporting of all suspected and confirmed cases to the local Public Health Unit is mandatory for consequent activation of vector control measures and active case finding. In addition, specific entomological surveillance is regularly performed when clinical cases of infection with DENV, Zika virus, chikungunya virus, West Nile virus or any other arboviruses are diagnosed. 

Previous undiagnosed autochthonous dengue cases in northern Italy cannot be excluded, especially if asymptomatic. A seroprevalence screening of the inhabitants of the village in Lodi province is ongoing in order to identify potential asymptomatic cases or recently infected people who had not travelled abroad. The extent of the outbreak may be defined only after the population screening. This study will be useful to reveal the extent of the DENV outbreak in this area, as reported by another study in Madeira [24]. Active clinical, epidemiological, virological and entomological surveillance is still ongoing in the area involved in dengue outbreak aimed at detecting further secondary cases. 

This report documented the first outbreak of DENV-1 in Lombardy region, highlighting the importance of continued integrated surveillance of imported virus infections and the potential for tropical disease outbreaks in the highly populated regions of northern Italy where *Ae. albopictus* has been present for many years [25]. Our findings suggest that the use of pan-flaviviruses PCR is fundamental for the differential diagnosis of the major pathogenic arboviruses belonging to the Flavivirus family. In parallel, virological surveillance of adult *Aedes* mosquitoes may be crucial for early identification of circulating arboviruses and rapid definition of local and regional public health measures that can control or prevent future outbreaks. 

## Conclusion

This preliminary report highlights the importance of continued integrated surveillance of imported vector-borne virus infections and the potential for tropical disease outbreaks in highly populated regions of northern Italy where competent vectors are present.

## References

[1] Bhatt S Gething PW Brady OJ Messina JP Farlow AW Moyes CL The global distribution and burden of dengue. Nature. 2013;496(7446):504-7. 10.1038/nature12060 23563266 PMC3651993

[2] Wilder-Smith A Ooi EE Horstick O Wills B. Dengue. Lancet. 2019;393(10169):350-63. 10.1016/S0140-6736(18)32560-1 30696575

[3] La Ruche G Souarès Y Armengaud A Peloux-Petiot F Delaunay P Desprès P First two autochthonous dengue virus infections in metropolitan France, September 2010. Euro Surveill. 2010;15(39):19676. 10.2807/ese.15.39.19676-en 20929659

[4] Gjenero-Margan I Aleraj B Krajcar D Lesnikar V Klobučar A Pem-Novosel I Autochthonous dengue fever in Croatia, August-September 2010. Euro Surveill. 2011;16(9):19805. 10.2807/ese.16.09.19805-en 21392489

[5] Alves MJ Fernandes PL Amaro F Osório H Luz T Parreira P Clinical presentation and laboratory findings for the first autochthonous cases of dengue fever in Madeira Island, Portugal, October 2012. Euro Surveill. 2013;18(6):20398. 10.2807/ese.18.06.20398-en 23410256

[6] Lazzarini L Barzon L Foglia F Manfrin V Pacenti M Pavan G First autochthonous dengue outbreak in Italy, August 2020. Euro Surveill. 2020;25(36):2001606. 10.2807/1560-7917.ES.2020.25.36.2001606 32914745 PMC7502902

[7] Navero-Castillejos J Benitez R Torner N Muñoz J Camprubí-Ferrer D Peiró-Mestres A Molecular characterization of imported and autochthonous Dengue in northeastern Spain. Viruses. 2021;13(10):1910. 10.3390/v13101910 34696340 PMC8539074

[8] Scaramozzino N Crance JM Jouan A DeBriel DA Stoll F Garin D. Comparison of flavivirus universal primer pairs and development of a rapid, highly sensitive heminested reverse transcription-PCR assay for detection of flaviviruses targeted to a conserved region of the NS5 gene sequences. J Clin Microbiol. 2001;39(5):1922-7. 10.1128/JCM.39.5.1922-1927.2001 11326014 PMC88049

[9] Linke S Ellerbrok H Niedrig M Nitsche A Pauli G. Detection of West Nile virus lineages 1 and 2 by real-time PCR. J Virol Methods. 2007;146(1-2):355-8. 10.1016/j.jviromet.2007.05.021 17604132

[10] Huhtamo E Hasu E Uzcátegui NY Erra E Nikkari S Kantele A Early diagnosis of dengue in travelers: comparison of a novel real-time RT-PCR, NS1 antigen detection and serology. J Clin Virol. 2010;47(1):49-53. 10.1016/j.jcv.2009.11.001 19963435

[11] Piralla A Borghesi A Di Comite A Giardina F Ferrari G Zanette S Fulminant echovirus 11 hepatitis in male non-identical twins in northern Italy, April 2023. Euro Surveill. 2023;28(24):2300289. 10.2807/1560-7917.ES.2023.28.24.2300289 37318763 PMC10318938

[12] Borges V Pinheiro M Pechirra P Guiomar R Gomes JP. INSaFLU: an automated open web-based bioinformatics suite "from-reads" for influenza whole-genome-sequencing-based surveillance. Genome Med. 2018;10(1):46. 10.1186/s13073-018-0555-0 29954441 PMC6027769

[13] Minh BQ Schmidt HA Chernomor O Schrempf D Woodhams MD von Haeseler A IQ-TREE 2: New models and efficient methods for phylogenetic inference in the genomic era. Mol Biol Evol. 2020;37(5):1530-4. 10.1093/molbev/msaa015 32011700 PMC7182206

[14] European Commission (EC). Commission Implementing Decision (EU) 2018/945 of 22 June 2018 on the communicable diseases and related special health issues to be covered by epidemiological surveillance as well as relevant case definitions. Brussels: EC; 2018. Available from: https://eur-lex.europa.eu/legal-content/EN/TXT/PDF/?uri=CELEX:32018D0945&from=EN#page=18

[15] Italian Ministry of Health. Piano Nazionale di prevenzione, sorveglianza e risposta alle Arbovirosi (PNA) 2020-2025. [National plan for prevention, surveillance and response to arboviruses (2020-2025)]. Rome: Ministry of Health; 2019. Italian. Available from: https://www.salute.gov.it/imgs/C_17_pubblicazioni_2947_allegato.pdf

[16] Becker N, Petric D, Zgomba M, Boase C, Madon M, Dahl C, et al. Mosquitoes and their control. Berlin, Heidelberg: Springer; 2010. Available from: https://link.springer.com/book/10.1007/978-3-540-92874-4

[17] Malavige GN Fernando S Fernando DJ Seneviratne SL. Dengue viral infections. Postgrad Med J. 2004;80(948):588-601. 10.1136/pgmj.2004.019638 15466994 PMC1743110

[18] Gubler DJ. Epidemic dengue/dengue hemorrhagic fever as a public health, social and economic problem in the 21st century. Trends Microbiol. 2002;10(2):100-3. 10.1016/S0966-842X(01)02288-0 11827812

[19] Cochet A Calba C Jourdain F Grard G Durand GA Guinard A Autochthonous dengue in mainland France, 2022: geographical extension and incidence increase. Euro Surveill. 2022;27(44):2200818. 10.2807/1560-7917.ES.2022.27.44.2200818 36330819 PMC9635021

[20] Romi R. History and updating on the spread of Aedes albopictus in Italy. Parassitologia. 1995;37(2-3):99-103. 8778671

[21] Romi R. [Aedes albopictus in Italy: an underestimated health problem]. Ann Ist Super Sanita. 2001;37(2):241-7. Italian. PMID:11758282 11758282

[22] Barzon L Gobbi F Capelli G Montarsi F Martini S Riccetti S Autochthonous dengue outbreak in Italy 2020: clinical, virological and entomological findings. J Travel Med. 2021;28(8):taab130. 10.1093/jtm/taab130 34409443 PMC8499737

[23] European Centre for Disease Prevention and Control (ECDC). Communicable disease threats report. Week 36, 3-9 September 2023. Stockholm: ECDC; 2023. Available from: https://www.ecdc.europa.eu/sites/default/files/documents/Communicable_disease_threats_report_3-9_September_2023_week_36.pdf

[24] Auerswald H de Jesus A Seixas G Nazareth T In S Mao S First dengue virus seroprevalence study on Madeira Island after the 2012 outbreak indicates unreported dengue circulation. Parasit Vectors. 2019;12(1):103. 10.1186/s13071-019-3357-3 30867031 PMC6417143

[25] Rovida F Percivalle E Campanini G Piralla A Novati S Muscatello A Viremic dengue virus infections in travellers: potential for local outbreak in Northern Italy. J Clin Virol. 2011;50(1):76-9. 10.1016/j.jcv.2010.09.015 21056002

